# Basic Income and Human Needs Satisfaction: Evidence from the HudsonUP Experiment

**DOI:** 10.1007/s11205-025-03708-5

**Published:** 2025-09-23

**Authors:** Nicholas Langridge, Leah Hamilton, Alex Dobill

**Affiliations:** 1 Department of Social and Policy Sciences, University of Bath, Bath, England; 2 Department of Social Work, Appalachian State University, Boone, USA

**Keywords:** Basic income, Post-growth, Eco-social policies, Sustainable welfare, Human needs

## Abstract

This article analyses qualitative data from the HudsonUP unconditional basic income (UBI) experiment to examine changes to participants’ human needs satisfaction. Human needs theories offer a holistic perspective on wellbeing and are widely employed in the sustainable welfare and post-growth literatures. However, they are under utilised in empirical UBI research. Through an inductive/deductive hybrid thematic analysis of interviews conducted at the baseline and three-year mark, the article examines changes in participants’ ability to satisfy their needs of subsistence, protection, freedom, participation, affection, leisure, understanding, creativity, and identity over the course of the experiment. In doing so, it demonstrates the viability of applying needs-based approaches to UBI research. Findings indicate that the participants’ ability to satisfy their material and non-material needs did increase over the course of the experiment. However, they continued to face barriers to full needs satisfaction. The findings suggest that cash alone is insufficient and proposals for an eco-social UBI – one which contributes to satisfying human needs within ecological limits – must also be accompanied by appropriate supply-side reforms. The article contributes to bridging the gap between theory and practice when it comes to the potential role of UBI in promoting socially just and sustainable welfare in line with post-growth perspectives.

## Introduction

Modern societies consider prosperity to be synonymous with material accumulation and economic growth (Jackson, 2017). Gross Domestic Product (GDP), which measures the monetary value of goods and services produced within an economy, is considered a proxy for human welfare within mainstream policymaking. At the individual level, wellbeing is often correlated to simply the quantity of material consumption (Raworth, 2017).

This material centred perspective on wellbeing is the subject of various critiques. GDP is considered a poor indicator of societal wellbeing (Daly, 1996; Giannetti et al., 2015; Hickel, 2019; Armstrong, 2022; Schmelzer et al., 2022) and, beyond a certain point, material accumulation does not enhance individual wellbeing (Easterlin et al., 2010; Alexander, 2012; Jackson, 2021). Despite huge economic growth over the past 200 years, billions of people still live below an “ethical” poverty line of around $5-a-day (Hickel, 2017) and social inequality has reached unprecedented levels (Piketty, 2015). Furthermore, there is now substantial evidence to suggest that the pursuit of aggregate economic expansion is incompatible with ecological sustainability (Parrique et al., 2019; Haberl et al., 2020; Vadén et al., 2020; Vogel & Hickel, 2023).

Alternative conceptions of wellbeing emphasise its non-material aspects, including the role of relationships, personal values, freedom and autonomy (Sen, 1999; Layard, 2011). The philosophy of ‘voluntary simplicity’ advocates for more fulfilling lives through the conscious choice to restrict material consumption (Alexander, 2011). Theories of human need take a holistic approach to wellbeing, including both its material and non-material aspects. Such theories recognise that all humans, across space and time, have certain objective and universal needs which can be met through an infinite variety of material and non-material satisfiers (Max-Neef, 1991; Gough, 2017).

Theories of human need have been widely employed within the growth-critical literature, which focuses on reducing the aggregate material and energy throughput of economies in line with ecological sustainability and social justice (D’Alisa et al., 2014)^1^. Needs approaches are also fundamental to ‘sustainable welfare’, a body of literature concerned with systems of welfare provision which meet the needs of all in line with planetary boundaries and without the need for economic growth (Büchs et al., 2024).

Unconditional Basic income (UBI) – the provision of unconditional cash transfers to all (Van Parijs & Vanderborght, 2017) – is frequently discussed within the sustainable welfare literature (Büchs, 2021). It is also one of the most widely cited policy proposals within the post-growth literature (Fitzpatrick et al., 2022). UBI is advocated for its potential to alleviate poverty, redistribute social wealth, change the nature of work, facilitate social and political participation and satisfy human needs in line with planetary boundaries (Langridge, 2024). However, there is a significant gap between such theoretical visions and the scope and focus of much of the empirical research. While human needs approaches are widely advocated within the post-growth literature (Gough, 2015; Büchs & Koch, 2019; Millward-Hopkins et al., 2020; Vogel et al., 2021) they are currently underutilised in the empirical research on UBI, which often focuses on more discrete metrics such as income or employment rates (Langridge et al., 2022; Langridge, 2024). There is an overall lack of empirical research on financial compensation policies, such as UBI, and needs satisfaction (Büchs et al., 2021). This is a major gap when considering UBI as a post-growth policy.

This article contributes to addressing this gap. Using an inductive/deductive hybrid thematic analysis, it examines data from the ongoing HudsonUP UBI experiment relating to changes to participants’ human needs satisfaction over time. The article makes two specific contributions. First, in line with post-growth perspectives, it verifies that needs-based approaches can be useful for analysing holistic wellbeing changes under UBI conditions and so begins to bridge the divide between post-growth theory and UBI practice. Second, the study provides insights into the potential for UBI to influence the depth and breadth of holistic wellbeing.

The following section provides an introduction to post-growth and sustainable welfare, theories of human need and UBI. Section 3 introduces the HudsonUP experiment and analysis methods. Section 4 outlines the findings before Section 5 discusses implications for UBI and human needs satisfaction. Section 6 offers some final conclusions. The findings suggest that the participants’ ability to satisfy their material and non-material needs increased over the course of the experiment, albeit at a gradual pace. However, participants continued to face barriers to full needs satisfaction given the experiment’s limited ability to influence the wider economy. The article highlights the limits to cash alone in achieving post-growth compatible wellbeing; cash provision needs to be supported by supply-side reforms as part of a wider package of sustainable welfare policies.

## Literature Review

### From Growth to Sustainable Welfare

Economic growth – as measured through GDP - is the key policy goal for modern economies (Jackson, 2017), deemed necessary for reducing poverty and improving living standards (UN, 2020). The pursuit of growth is, however, critiqued on ecological and social grounds. From an ecological perspective, the evidence is overwhelming that aggregate growth is incompatible with planetary boundaries (Parrique et al., 2019; Haberl et al., 2020; Hickel & Kallis, 2020; Vadén et al., 2020; Vogel & Hickel, 2023). From a social perspective, GDP excludes many activities which contribute to social wellbeing (e.g. unpaid care work) and includes activities which are actively destructive (e.g. war, smoking and environmental pollution) (Van den Bergh, 2009). Furthermore, the majority of the benefits from growth go to the already wealthy: 38% of new wealth created since the 1990s has been captured by the richest one percent of the population (Chancel et al., 2022) where it contributes little to furthering wellbeing (Jackson, 2017). These critiques have led to the emergence of post-growth movements and academic areas of scholarship which question the focus on growth and instead pursue an equitable downscaling of the material throughput of the economies in line with social justice and ecological sustainability (Islar et al., 2024).

The sustainable welfare literature – a sub-section of the wider post-growth movement - focuses on systems of eco-social policies that support human wellbeing in line with planetary boundaries and without the need for further growth (Büchs et al., 2024). Büchs (2021, p.2) identifies four “essential principles of sustainable welfare”, including respect for planetary boundaries, fair distribution of resources and opportunities, democratic governance and the satisfaction of human needs. This article examines UBI’s compatibility with the last of these principles through an analysis of data from the HudsonUP experiment.

### From Means-based Prosperity to the Satisfaction of Human Needs

When social progress is believed to correlate with expanding material production and consumption, development is reduced to the process of simply increasing the quantity of available material means (Patnaik, 2010). Material accumulation is considered a synonym for development, prosperity and wellbeing (Patnaik, 2010; Raworth, 2017; Jackson, 2021).

Prioritising material production and consumption increases may make sense where the resources for living a good life are lacking but, beyond a certain point, equating quantity with quality, or more with better, is misguided (Easterlin et al., 2010). The Genuine Progress Indicator (GPI) is a metric which addresses the “shortcomings of GDP by measuring the social and environmental costs and benefits which GDP either ignores or counts as economic progress” (Anielski & Daly, 2007, p.32). Despite GDP growing more or less continuously prior to the 2008 financial crisis, the GPI in high-income nations levelled off in the 1970s before beginning to decline (Jackson, 2017). The point at which GPI deviates from GDP can be interpreted as the point at which the social and environmental costs of growth cancel out consumption-related gains. Post-growth scholars therefore reject wellbeing theories centred on increasing the aggregate *quantity* of material resources, at both the societal and individual level, in favour of those focused on the *quality* of material resources and the intangible, non-material factors (Jackson, 2021).

Human needs approaches provide a holistic understanding of wellbeing and are often employed in the sustainable welfare and post-growth literatures (Gough, 2015; Büchs et al., 2024). Such approaches recognise that “all individuals, everywhere in the world, at all times present and future, have certain basic needs” which are “objective,” “plural” (i.e. cannot be aggregated), “non-substitutable,” “satiable” and “cross-generational” (Gough, 2017, pp.42–48). Max-Neef's (1991) work on Human Scale Development identifies a framework of nine fundamental human needs (FHNs): subsistence, participation, freedom, protection, affection, idleness, creation, understanding and identity. While these needs are universal, they can be met through a potentially infinite number of “satisfiers” which include goods, services, relationships and activities relevant to different contexts, cultures or times. For example, the need for subsistence can be met by an endless variety foodstuffs and cuisines. A satisfier that meets more than one need is a “synergic satisfier.” Conversely, one which hinders the satisfaction of other needs is an “inhibitor” or “violator”.

Unlike wants or desires, human needs are satiable and non-substitutable and so allow the minimum material requirements for wellbeing to be defined and provide a focus for material production and consumption (Gough, 2017). Needs theories are also rooted in ecological sustainability (Guillen-Royo, 2018). Considering needs across space and time requires the maintenance of the natural world so that future generations can satisfy their own needs (Doyal & Gough, 1984). Furthermore, by widening the scope beyond material consumption needs approaches encompass non-material satisfiers such as enhanced relationships, more free time and changing values (Kallis et al., 2020). The FHN framework was developed on the premise of a “reasonable use of resources that a person needs to have an acceptable quality of life” and therefore bring human development back into harmony with nature (Caria & Domínguez, 2019, p.55). By focusing resources on the satisfaction of satiable needs, including through non-material satisfiers, human needs approaches remove the requirement for economic activity which does not contribute to wellbeing. For these reasons, needs approaches are favoured by many post-growth scholars (Gough, 2015; Büchs & Koch, 2019; Millward-Hopkins et al., 2020; Vogel et al., 2021).

### Unconditional Basic Income for the Satisfaction of Human Needs

UBI is usually defined as a “a regular cash income paid to all, on an individual basis, without means test or work requirement” (Van Parijs & Vanderborght, 2017, p.1). The *universality* aspect (i.e. that it is paid to all), however, is not always present in experimental research; practical limitations often prevent UBI ‘pilots’ from making universal payments (Widerquist, 2018). Furthermore, variants such as the Minimum Income Guarantee (MIG) or Guaranteed Income (GI) (a term more common in the USA) may target payments to those on low incomes, with the view to ensuring that no one falls below a minimum standard (UBI Lab Network, 2021). The *unconditional* aspect, in the sense that there are no conditions placed on how people can use the cash, however, remains constant. Such unconditionality is considered a fundamental and defining feature of all forms of UBI (Widerquist, 2013; Van Parijs & Vanderborght, 2017; Casassas, 2024). UBI is used as a collective term for all unconditional cash transfer experiments in this article.

UBI is advocated as a policy for reducing poverty, deprivation and inequality (Lowrey, 2018). A meta-analysis conducted by the Stanford Basic Income Lab found evidence that UBI “leads to a measurable decrease in poverty” in low- middle-income countries. However, evidence in “advanced economies” is more limited and underexamined (Hasdell, 2020, p.15).

In the USA, over 150 UBI/GI pilots have launched since 2017 (Elliott et al., 2023). However, because this growth has been largely organic — driven by a mixture of local government, non-profit and philanthropic initiatives, many of which were launched rapidly in response to COVID-19 using American Rescue Plan Act (ARPA) funds — few of these programmes have used experimental designs or been evaluated in peer-reviewed publications (Okantey, 2024).

Nonetheless, a small but growing number of systematic reviews and meta-analyses have begun to synthesise findings from those pilots with rigorous evaluation designs in the USA and other high-income countries. Rizvi et al. (2024) found moderate evidence that UBI initiatives reduced food insecurity and improved mental well-being and life satisfaction, especially when provided as a supplement to existing welfare. Nishimura et al. (2025) demonstrated similar benefits for mental and financial well-being. Calhoun et al. (2024) reviewed studies focused on cash benefits for people experiencing homelessness in the USA and found improvements in housing, food security and quality of life. Brydon et al. (2024) conducted a broad scoping review of experimental studies across high-income countries and reported that about 75% found beneficial effects on health outcomes. Together these reviews offer growing, though still partial, evidence that UBI can alleviate poverty and improve health and social outcomes in high-income settings.

UBI features prominently within the sustainable welfare literature (Büchs, 2021) and is one of the most cited policies within the post-growth literature more broadly (Fitzpatrick et al., 2022). Advocates argue that UBI has the potential to improve wellbeing within ecological limits. Specifically, it could provide people with the time and resources to reject situations that inhibit or violate the satisfaction of their needs, such as engaging in unnecessary production and consumption, exploitative labour or abusive relationships and instead engage in activities which do, such as food production, care work, volunteering, social participation or collective political action (Langridge, 2024).

However, support for UBI is not universal. Critics argue that its reliance on the market for providing goods and services could reduce the role of the public sector (Coote & Percy, 2020). In an economy focused on production for exchange and profit rather than production for use, there is no guarantee that markets would provide (only) the goods necessary for satisfying needs and in the most ecologically sustainable way. Other concerns include UBI’s reliance on the state (Paterson, 2016; Buch-Hansen & Koch, 2019), the lack of evidence to support UBI facilitating an “exit” from work activities which do not satisfy needs (Birnbaum & De Wispelaere, 2016), its inherent freedom which offers the opportunity to make consumption decisions which do not align with needs satisfaction or ecological sustainability (McGann & Murphy, 2023) and scepticism regarding the role of cash transfers in overcoming gendered (and other) inequalities inherent to care work (Orloff et al., 2013; Lombardozzi, 2020; Bärnthaler & Dengler, 2023).

There is a distinct lack of empirical research that considers UBI’s impact on human needs satisfaction (Büchs et al., 2021; Langridge, 2024). UBI experiments often adopt more discrete wellbeing metrics which align the pursuit of growth (Langridge, 2024). This is an important gap which contributes to the lack of empirically researched, post-growth UBI proposals (Parrique, 2019).

## Methodology

This article analyses data from the HudsonUP experiment^2^ using Max-Neef’s framework of fundamental human needs. It seeks to answer the following research question: *How does the ability of participants to satisfy their needs change under the conditions of the experiment?* In answering this question, the article seeks to demonstrate how needs-based frameworks of wellbeing can be employed in UBI research.

### The HudsonUP Experiment

In 2020, non-profit leaders in the city of Hudson, New York, announced a five-year experiment for adult residents living on less than the area’s median income. Participants would receive $500 per month for five years, a total of $30,000. In alignment with the principles of UBI, the experiment placed no obligation on how participants use the money; the cash payments were unconditional. However, the payments were not universal. Potential participants were informed about the experiment through flyers, community meetings, and social media channels. Only those living below the city’s median income level were eligible to apply, with the final selection based on a randomised sample weighted by race, ethnicity, gender and income, in keeping with the programme’s commitment to equity (For more detail see Hamilton, 2021). In October 2020, 25 applicants were selected to participate in the first cohort. Subsequent lotteries added a second and third cohort, in 2021 and 2022 respectively. Following selection, participants were invited to also participate in the research component. They were informed that rejecting the invitation would not affect their payments. Ultimately, 15 participants of the first cohort chose to participate in the research process.

### Data Collection

All participants expressing interest in the research component were invited to participate in open-ended, semi-structured interviews, conducted either in person or via phone according to preference. This ensured participant comfort and encouraged open, candid discussions. The in-person interviews were held in quiet, private settings to create a safe and comfortable environment. Each interview was designed to last approximately one hour, providing enough time for participants to explore their experiences in depth. The semi-structured format allowed for a guided yet flexible conversation, with room for participants to express thoughts and stories beyond the guiding questions (Yin, 2015). The content of the interviews revolved around participants’ experiences of economic changes, personal and family wellbeing, community interactions and perceptions of the future but did not specifically refer to any needs-based framework. Each interview was transcribed verbatim and anonymised. Completed surveys and interviews were compensated $20, chosen to correspond with the median hourly income in the area and so fairly compensate participants for their time. The data analysed in this article comes from the qualitative interviews undertaken at the baseline and three-year stage with all 15 participants of the first cohort who took part in the research component.

### Data Analysis

The article employs an inductive/deductive hybrid thematic analysis (Fereday & Muir-Cochrane, 2006; Proudfoot, 2023). Inductive thematic analyses allow themes to emerge organically from the data (Boyatzis, 1998) and helps “ensure that the voices of the participants are valued” (Proudfoot, 2023, p.309). Deductive analyses apply an explicit theoretical framework to the data (Crabtree & Miller, 2022) and so allow for more “theory-led analysis” (Proudfoot, 2023, p.309). Applying an inductive/deductive hybrid approach to the same dataset can increase rigour and harness the advantages of each (Fereday & Muir-Cochrane, 2006; Proudfoot, 2023, p.309).

The interview transcripts were first subjected to an inductive thematic analysis, informed by Schutz (1967)’s theory of social phenomenology. Social phenomenology emphasises participants’ *subjective* perceptions of their life experiences. It therefore prevents “social reality” from being replaced by a “fictional, nonexistent world constructed by the researcher” (Fereday & Muir-Cochrane, 2006, p.81). The research team conducted an initial reading of the transcripts and used Taguette software to code statements relating to participants wellbeing and subjective experiences during the period of the experiment. These statements were then grouped into themes. At each wave of data collection, themes and representative statements were shared with participants for member checking (Lincoln & Guba, 1985). The inductive approach aimed to authentically collect the subjective experiences of the participants. The analysis team were not informed of the upcoming deductive analysis phase nor of the role of the FHN framework during this process.

The themes generated through the inductive analysis were then mapped against Max-Neef's (1991) framework of fundamental human needs (see section 2.2). The FHNs framework offers a typology of needs which provides a holistic approach to understanding wellbeing, including the material and non-material aspects. It is therefore considered an appropriate framework for examining wellbeing in post-growth contexts, as discussed above. The intention of the inductive analysis was to examine how closely the conceptions of wellbeing articulated by the participants themselves aligned with the FHN framework and so determine its credibility. It also helped demonstrate which needs were most present in the participants’ experience at different stages of the intervention.

The research team then re-analysed the transcripts deductively, using the FHN framework as a theoretical guide. The researchers re-read the transcripts and used NVivo to code specific examples of where the participants spoke to the various needs in the framework and discussed how they were able (or unable) to satisfy them at the different stages of the intervention. This approach is similar to other studies employing FHN as a theoretical framework (e.g. James, 2021).

By focusing on the first cohort, the analysis was able to capture how the experiences of the participants changed over time and through different stages of economic security (Saldaña, 2003). This is particularly relevant given the dynamic nature of economic and social conditions and enables a richer, more comprehensive understanding of any changes. The participants were not asked directly if they believed the cash had caused any changes. Instead they were simply asked to explain what had changed in their lives and were free to assign their own perceived causation. By employing a constructivist lens, we acknowledge that these experiences are not just individual but are also shaped by broader social, economic, and cultural contexts.

### Ethical Considerations

Ethical considerations were paramount to ensure the integrity and responsibility of the study. The study received ethical approval from the Appalachian State University Institutional Review Board in 2020. Participants were provided with information about the study, including its purpose, the nature of their participation, potential risks and any benefits. They were informed of their right to withdraw at any point without negative consequences. The consent form was made available in clear, understandable language and ample opportunity was given for participants to ask questions. All personal identifiers were removed and pseudonyms were used when discussing or presenting the data. Secure data storage methods were implemented to protect the data from unauthorised access. Recognising the potential power imbalances between the research team and the participants, efforts were made to approach the research relationship with humility and respect.

## Findings

### Mapping Themes to Fundamental Human Needs

Table 1 presents the themes which emerged from the inductive thematic analysis of the interview transcripts at the baseline and three-year stages. They provide insight into the participants’ experiences and changing perceptions, feelings and responses to life situations over the course of the experiment. In column three, the themes have been mapped against the FHN framework. Part of the intention here is to demonstrate the credibility of the framework.

**Table 1:** Mapping themes from inductive thematic analysis to FHN framework

Themes emerging from inductive thematic analysis		Fundamental Human Needs
Baseline (Wave 1)	Three-year point (Waves 5 & 6)	
Security (Financial, housing, and vulnerability to shocks) Traditional Public Assistance	Health (Physical and mental) Security (Financial and emotional)	Subsistence
		Protection
Agency Life in Hudson (Transport)	Making Choices Financial independence	Freedom
	Perspectives on Poverty Personal Growth (Education)	Understanding
Life in Hudson (community) Relationships	Community Connections Reduced Societal Judgement Relationships Generosity	Participation
		Affection
Personal Goals	Personal Growth (Investing in themselves)	Creativity
		Identity
	Leisure	Leisure
Feelings Regarding Experiment	Positivity About BI	
	Concerns for Programme End	

Most themes emerging from the inductive analysis could be mapped to at least one of the needs in the FHN framework. This suggests a general alignment between the normative understanding of wellbeing presented in the FHN framework and the subjective experiences of participants.

The table also suggests that there were improvements in holistic wellbeing over the course of the experiment. While the themes emerging from the transcripts during the baseline could be mapped to some needs in the FHN framework, several remained un-, or under-addressed (leisure, understanding, identity, creativity). Themes from the three-year point could be mapped to a greater number of FHNs: Participants elaborated further on needs under-addressed in the baseline transcripts (identity, creativity, participation, affection) and also discussed those which were previously absent (leisure, understanding).

Some themes emerging from the analysis did not map directly to the FHN framework. These included feelings about UBI as a policy, the experiment itself and concerns regarding its end. However, these were intervention specific and not relevant to holistic wellbeing.

### Findings from the Deductive Thematic Analysis

The findings discussed below emerged from the second, deductive analysis of transcripts against the FHN framework. While presented under separate headings, this is an artificial distinction as there is clear overlap and interaction between the needs and satisfiers. Pseudonyms have been used to protect the participants’ identities.

### Subsistence

#### Baseline

Subsistence was the most widely discussed need in the baseline interviews. Many participants reported being unable to satisfy their basic subsistence needs, citing insufficient access to food, housing and healthcare. Those that could meet their subsistence needs did so with difficulty. Only two participants said that they did not struggle in this regard.

Access to housing was the most widely discussed satisfier of the need for subsistence. Many participants reported supply issues and unaffordable costs. Sofia explained: *“There’s no feasible living situation for me here anymore…because of both housing stock and prices…I am afraid every day of losing my apartment.”* Maria said *“the housing [is a disadvantage]*,* how high the rent is and you can’t find no apartment.”*

Alongside a lack of affordable housing, other key inhibitors to the satisfaction of subsistence needs were poor health (either of participants themselves or those for whom they were caring) and mobility issues, i.e. a lack of access to transportation. Both prevented participants from accessing the economic goods and/or paid work necessary for satisfying their subsistence needs.

#### Three-year point

At the three-year point subsistence was still the most discussed need. However, all participants reported improvements in their ability to satisfy it. Nia shared: *“I was able to do everything*,* everything was taken care of*,* so that was a relief.” *Similarly, Sofia said *“I don’t have to think about making rent.”* Aisha said *“I’ll tell you something*,* I’m stressed out*,* but I would be much more stressed out without this $500 that I just know is coming every month. Because I just know*,* okay*,* certain bills and my food is taken care of and it really does make a huge difference in my mental being a little calmer.”*

However, the lack of affordable housing was an ongoing issue for some participants. Fatima explained: *“The cost of everything has gone up and it’s so difficult to find housing”* while Sofia claimed *“We are paid better than other people*,* but not enough to live in Hudson.”* While most participants reported general stability in their housing situation, challenges remained. Housing prices had forced participants to find alternative living arrangements, including staying with family. Several reported uncertainties as to whether they would be able remain in their current homes.

Health issues - for both participants and their families - also continued to inhibit the satisfaction of subsistence needs at the three-year point. While improvements to both mental and physical health were reported, ongoing challenges included healthcare costs, the time and logistical requirements for accessing treatment and the emotional stress poor health entails. Several participants reported using the cash for medical and care expenses. Nia explained: *“Right now*,* my priority is my husband. With him being in a nursing home*,* they take money. So*,* it really came in handy because it gave me a little cushion.”* Fatima mentioned that they could now take sick days: *“I’m feeling sick today*,* my son is feeling sick today*,* I can’t go to work. So*,* I don’t have that source of income coming in. So*,* [the cash transfer] is constant and it’s sure. It has empowered and has changed my life”*. Several participants mentioned that they now had access to some form of health insurance.

### Protection

#### Baseline

Protection was also frequently discussed during the baseline interviews. The most cited satisfiers of this need at the start of the intervention were financial security and savings (or a lack thereof), shelter and housing and health insurance.

Many participants reported never being able to save money and having limited financial security. A couple mentioned difficulties resulting from unexpected health shocks. A key driver of financial insecurity was the high proportion of income spent on satisfying subsistence needs. As mentioned above, a lack of affordable housing was a key challenge to satisfying the need for protection for many participants.

Participants expressed a desire to save and be more financially secure and cited concerns about future ill-health or old age. The lack of financial security increased participants’ stress and contributed to poor mental health.

#### Three-year point

The desire for financial security, including reduced debt and putting money into savings, continued to feature during interviews at the three-year point. James said,*“I think number one would be to eradicate my high interest debt. So*,* student loans or if I have any credit card debt.”* Aisha hoped *“I can start saving a little bit of that again.”* While several participants still faced obstacles to saving, including a lack of knowledge and living paycheck-to-paycheck, several did report that they had been able to turn their desire to save into reality. Aisha continued *“Finally last year I was able to open a Roth IRA for the first time so that I can start contributing to my future in some way.”*

Several participants mentioned that they felt they had more stability when it came to work and housing. Sofia explained: *“I’m feeling very secure and stable and good right now…I don’t have to think about making rent.”* Aisha said *“I am in the same place and I’m so grateful for that because I do really like my setup in my apartment. And it’s the one thing that one would call a safe space”.* However, rent increases, as discussed above, did add to participants’ sense of insecurity, with housing prices forcing people to find somewhere else to live or creating uncertainty.

### Freedom

#### Baseline

Freedom was the third most referenced need at baseline. The most widely discussed satisfiers included access to physical mobility, (lack of) control over life choices and the impact of poor health on freedom.

With regards to mobility, participants regularly noted the need for a car to access their work, shops and other necessary services, particularly if they lived outside the city centre. Several participants relied on friends and family to access such services. The lack of access to transport restricted the freedom of participants and their ability to satisfy other needs.

Health conditions created barriers to some participants’ ability to work, thereby restricting the control they felt like they had over their lives and limiting their access to resources for satisfying other needs.

Participants also reported a lack of independence, a reduced choice of work opportunities, an inability to do or buy what they wanted and a lack of ability to invest in their futures. Paula discussed how she was unable to leave a toxic relationship prior to the project. While such factors speak to an inability to satisfy the need for freedom, they also speak to other needs within the FHN framework.

#### Three-year point

At the three-year point, many participants perceived improvements in their ability to satisfy the need for freedom, largely due to increased free time and choice over how to use their money. Sofia said *“I have a more clear mind and I can spend my free time being free.”* Nia asserted that *“when it comes to my money*,* you know what*,* it’s my money… I was told this is mine to do whatever I want.”* However, some participants mentioned feelings of judgement or guilt that came with increased financial freedom. Sofia mentioned wanting to keep the money secret so that people didn’t think of them as a *“privileged middle-class kid.”* Nia expressed feeling responsibility over how to use the money: *“These people afforded me this. Do I really want to blow their money?”*

A lack of mobility continued to inhibit full satisfaction of the need for freedom for several participants who, despite having the desire to be more mobile, had not been able to make this a reality. Emma explained: *“I really didn’t need that car at one time. But I need one now… I’ve been working on it hard*,* but I haven’t succeeded yet.”* James described how he had a 70-minute commute comprised of walking and riding the train.

### Participation

#### Baseline

Hudson’s strong sense of community was the most commonly mentioned satisfier of the need for participation. Jessica explained: *“I love the community. Everybody works together and tries to help each other.”* Lena shared: *“It’s where everybody wanted to be during quarantine*,* and I was already here. Tight community.”* However, Amara and Denise mentioned racial divides inhibiting satisfaction of their need for participation: *“It’s hard living here having bi-racial kids…I get it from both sides”* and *“The disadvantages are opportunities for minorities to be part of the community*,* outside their segregated streets.”*

The participants’ ability to satisfy the need for participation was also inhibited by a lack of financial resources, which affected their social lives. Some were also restricted by a lack of transportation or because of health conditions (as discussed above).

#### Three-year point

During the latter transcripts many participants continued to express positive feelings about the community. Nia said *“Hudson is my home. I love being in Hudson…The community has been so good to me and you realise all that when someone dear to you passes away. I had so much support.”* Nia further explained how community participation had helped overcome mobility issues: *“I am just blessed that I can pick up my phone and be like*,* ‘Oh*,* I need a ride here. I need to go here’ and I got somebody right there to help me”*. However, others reported negative changes, including the closing of stores due to higher rents.

Some participants expressed that the intervention created a desire to give back to their community. Emma commented: *“I say it from the heart. [HudsonUP] really helped me to help others because…I guess my overall thing I want to do is help others*,* and that’s the truth…hopefully*,* if I’m able*,* I’ll be able to do something like this*,* be able to be a part of helping people.”* Fatima explained: *“So yeah*,* maybe I really need to look into being an immigration attorney for real*,* to really help some people to go through all their stress.”*

### Affection

#### Baseline

The need for affection was discussed less frequently than those for subsistence, protection and freedom but considerably more than the needs for leisure, understanding, creativity and identity, which are discussed below.

The recurring factor affecting satisfaction of the need for affection was a reliance on family and friends for financial support to satisfy basic subsistence needs. John explained: *“I get by from the help of some people. I’ve got a close family.” Aisha added*,* “I’m very lucky to have a lot of great family and friends where I know I have a support group*,* so that’s huge.”* Participants noted the strain this placed on their relationships, causing arguments and mental stress. Lena shared: *“I’m needy towards friends*,* and that hurts relationships.”* Amara explained that *“[finances] took a toll on us… Maybe it causes more stress between us*,* but I don’t like to project stuff like that on my kids.”* Prior to the project, the strain imposed by a reliance on others affected participants' ability to satisfy the need for affection.

#### Three-year point

Financial reliance on friends and family continued to feature in the latter transcripts. James explained, *“I actually moved in with my family in Long Island…Now I get to stay with my parents and my brother and his family.”* However, Aisha mentioned being able to reduce this reliance: *“My family and friends are not a bottomless pit of borrowing. This has made a huge difference…Through the pilot now*,* I don’t have to worry about paying rent or asking my family to help me with paying rent.”* Participants also mentioned improved relationships with family and friends. Nia said *“I just like being around my family*,* and just doing things*,* and they have been awesome.”* James explained: *“It’s been really nice*,* actually having dinner with your parents and stuff.”*

In addition, participants mentioned caring responsibilities more frequently during the latter transcripts. While this demonstrated affection, it also created difficulties in life-balance and making time for themselves. As above, participants also expressed affection for their community and a desire to give something back.

### Leisure, understanding, creativity and identity

#### Baseline

The remaining needs from the FHN framework (leisure, understanding, creativity and identity) received far less attention at baseline. Where participants did speak about the need for leisure, they generally noted a lack of resources available for leisure activities. However, access to nature was given as an advantage of living in Hudson.

The need for understanding was only mentioned with regards to formal education. Several participants intended to use the money for this purpose. Regarding creativity, two participants mentioned the desire to use the money to start businesses, in car bodywork and cooking. Aisha mentioned how financial struggles affected their creativity as an artist.

With regards to identity, two respondents mentioned racial identity and some of the inequalities present in Hudson. Several wished to be free from a reliance on others for meeting their subsistence and protection needs and one expressed the desire to be a homeowner.

#### Three-year point

Leisure, understanding and creativity were all more frequently referenced in the latter transcripts. For example, James discussed the leisure activities that he was now able to do: *“Me and my friends*,* this summer*,* we went to Colorado for a camping trip*…*I’ve been getting into swimming now and a little bit of yoga”.* Sofia “*could take a Friday off and go do something and not worry about making rent for taking a day off.”* However, many still struggled to find the time to fully satisfy this need. A key reason was spending more time in wage-labour. Omar explained: *“I still do music*,* albeit less since I’m focusing on work. I’m usually exhausted after a day of work”*. Similarly, Sofia described how she had *“not really had the energy to do anything outside of work… I’m working more so my time off work*,* I like to just not do anything.”*

Formal education still dominated satisfaction of the need for understanding. However, some participants mentioned that they were also satisfying this need through their work. James said, *“I’m learning so much and I actually feel like I’m developing some clinical skills and gaining meaningful interactions with the patients.”*

Several participants in the latter transcripts displayed more ability to satisfy the need for creativity. For example, Nia shared: *“It also has allowed me to really open up my mind for my writing”.* Emma was planning to write a book which would include her experiences of the experiment and had also opened a catering business with her nephew. A further two other participants had also made steps towards starting their own businesses. Aisha discussed how the project had helped her as an artist.

The need for identity was still largely unaddressed in the latter transcripts. Fatima mentioned the desire to have a job which reflected her qualification level. Conversely, Sofia expressed a fear that receiving the cash challenged her working-class identity.

## Discussion

The data analysed in this article comes from a relatively small intervention in a particular socio-economic context. The generalisability of findings is therefore limited. However, the analysis demonstrates the applicability of normative human needs approaches to empirical research on UBI and holistic wellbeing and also provides some insight into changes to needs satisfaction under UBI conditions. While many of the specific findings may not come as a particular surprise, the article is the first – to the authors' knowledge - to empirically examine changes to holistic wellbeing under UBI conditions by employing a human needs approach.

The findings demonstrate tangible, albeit subtle, changes to human needs satisfaction among participants over the course of the HudsonUP experiment. The participants appeared to increase their satisfaction of needs which were previously under-addressed while also beginning to satisfy, or at least recognise, other previously neglected needs. However, improvements were subtle, highlighting the limitations of providing cash alone within the wider socio-economic context.

The needs for subsistence, freedom, protection, participation and affection were the most discussed by participants at baseline although in many instances participants mentioned their *inability* to satisfy them. This was expected given the low socio-economic status of the participants. However, as time progressed, participants began to discuss these needs more positively. Participants spoke of improvements in access to food, health and shelter (subsistence); plans, if not concrete actions, for improving financial and housing security (protection); more free time and a perception of autonomy over their decisions (freedom); and improved family and community relationships, including the desire to participate and give back to the community (participation and affection). The themes emerging from the inductive analysis mapped most clearly to these five needs and became clearer at the three-year point, particularly for the needs of participation and affection, suggesting greater depth to discussions regarding these needs in the latter transcripts.

The latter transcripts demonstrated linkages between different needs. Improved satisfaction of the needs for subsistence and protection, for example, helped improve satisfaction of the needs for affection and participation. Reductions in health and financial stress, and a reduced reliance on others, appear to have improved participants’ relationships with family, friends and the wider community. This suggests that the unconditionality of the cash could have multiplied benefits.

Second, the themes from the inductive analysis mapped to a greater quantity of needs at the three-year point than at the baseline. This suggests that participants’ concept of wellbeing became more holistic as the experiment progressed. This was supported by the second round of analysis. During the latter transcripts, the needs for leisure, understanding and creativity were given more attention by participants. For example, some mentioned going on trips, undertaking activities with friends or having more free time (leisure); others mentioned investments in education and skills and demonstrated a greater awareness of poverty (understanding) and a couple had started their own businesses (creativity). However, while participants spoke to these needs in the latter transcripts, many were aware of limitations in their ability to fully satisfy them. For example, several participants mentioned that satisfying their need for leisure was restricted due to spending more time in education or wage-labour. In this way, wage-labour, while contributing to the satisfaction of the needs for subsistence and protection, could also be seen as an inhibitor of the need for leisure. The direct contribution of the cash to time spent on these activities is unclear although some participants did mention using the cash to retrain or start a new business.

While the FHN framework presents all nine needs as important for holistic wellbeing, the findings suggest some take greater priority than others, with the more material needs of subsistence and protection taking precedence over those of leisure, creativity and identity. This aligns with other needs frameworks, such as Maslow’s hierarchy (McLeod, 2007) or Kaufman's (2021) sailboat. As participants improved their satisfaction of these more material needs, albeit in small and gradual ways, other, less material needs came more into their consciousness.

Third, while the provision of a UBI has the potential to impact demand, its impact on supply is more limited. While recipients may be able to make changes to the scale and composition of their consumption, their choices remain limited by the wider availability of goods and services. This is evidenced in some of the findings from this study. Despite receiving cash transfers for three-years, participants still regularly discussed issues with housing. While the UBI helped with the payment of rent, the cost and availability of housing remained an issue which inhibited participants’ ability to satisfy their needs for protection, subsistence and freedom. The same was true of access to healthcare. Similarly, improving mobility was a priority for several participants. This was important for satisfying the needs of subsistence, participation and affection, for example, by accessing work and basic goods or visiting friends and family. However, for many, their only option was to use the cash to access private transport options, such as buying or leasing a car. This has negative ecological implications and does not align with a degrowth transition. Participants also noted a lack of free or low cost public leisure spaces and activities. When financial resources were most scarce (i.e. at the baseline) this need remained unsatisfied, as discussed above. However, as the UBI increased people’s purchasing power, and they were able to give the need for leisure more attention, the lack of community leisure activities forced them to satisfy the need through material consumption. Again, this has negative ecological implications. Improving needs satisfaction in line with ecological sustainability therefore requires UBI to be accompanied by additional reforms which impact production, or the supply side of the economy.

The findings discussed in this article are subject to several limitations. First, it is impossible for UBI experiments, such HudsonUP, to pilot a full UBI. Like many such studies, the experiment provided cash to only a small section of the community, for a limited period, and did not include a scalable funding source. Such limitations are common to UBI experiments (Widerquist, 2018) and therefore require the empirical research on UBI to be supported by additional and alternative methods, such as laboratory experiments, microsimulations or explorative qualitative methods (Langridge, 2024). Second, as mentioned above, the cohort examined was relatively small, limiting the generalisability of findings. The study also lacked a control group, making the findings the findings more correlative than causative, although the presence of latent counterfactuals can provide some insight into possible causality (Copestake et al., 2019). Third, the findings come from interviews conducted using inductive methods. In other words, the interview questions did not refer to human needs frameworks specifically but were open and allowed participants to discuss their own experiences and priorities. Therefore, the questions asked during the baseline were different to those asked at the three-year point. This makes direct analysis of changes to needs satisfaction over time more difficult. However, it also gives a more holistic overview of lifestyle changes and the needs which were most prominent in participants’ minds at any given time. Finally, some participants mentioned changes in their social participation seasonally. During the summer, they mentioned that they had more opportunities/motivation to be social or participate in their communities. The time of the year in which interviews were conducted could therefore have impacted some responses.

## Conclusion

The dominant discourse links wellbeing to economic growth and aggregate material accumulation. This is strongly challenged from both social and ecological perspectives. Alternative visions embrace more holistic perspectives of wellbeing with resources targeted towards the meeting of human needs and greater attention paid to the intangible aspects. Human needs theories are often favoured within the post-growth and sustainable welfare literatures.

UBI is one of the most cited and discussed policies within these literatures. However, its support is not universal and significant knowledge gaps remain, including on its ability to increase holistic wellbeing while limiting unnecessary material consumption.

This article analysed qualitative data from the HudsonUP UBI experiment to provide insight into human needs satisfaction changes under UBI conditions, guided by Max-Neef's (1991) FHN framework. Human needs approaches are currently underutilised in the empirical UBI research, limiting a more rigorous and empirically informed examination of UBI as a post-growth policy. The article has demonstrated that needs-based approaches can be useful for analysing holistic wellbeing changes under UBI conditions and so begins to bridge the gap between post-growth theory and UBI practice.

The findings suggest that needs satisfaction increased over the course of the intervention, albeit at a gradual pace. They also demonstrate that the intervention widened participants’ view of wellbeing, as more needs from the FHN framework were discussed as the project developed, even if participants were not yet able to fully satisfy them. This suggests that the long-term provision of unconditional cash can improve needs satisfaction, albeit in small, gradual ways. It also suggests that, initially, certain needs take greater priority than others and it is only when these high priority needs are better satisfied that attention turns to others.

However, the findings also demonstrate the limitations of UBI. Some of the satisfiers employed by participants prioritised private, material consumption over more collective approaches, aligning with continued, aggregate economic growth. This was largely due to the limited options available in the wider economy which remain unaffected by the provision of a UBI, at least at the scale of the HudsonUP case study. However, the provision of cash made such limitations more bearable for participants. As part of a wider package of eco-social policies for sustainable welfare, therefore, UBI needs to be accompanied by supply side reforms. The impact of unconditional cash transfers for social and ecological wellbeing will depend on the context of the wider society into which they are introduced.

## Data Availability

Data available on request due to privacy/ethical restrictions.

## Footnotes

1 Hereon referred to as the post-growth literature, due to the focus on the transition to, and composition of, steady-state societies that do not pursue aggregate economic growth.

2 Torry (2023) argues that only projects testing a true UBI across a representative population and funded by tax revenue can be referred to as ‘pilots’. Given the ‘practical impossibility’ of testing a true, national-scale, universal UBI (Widerquist, 2018, p.37) the term ‘experiment’ is preferred to pilot to describe the HudsonUP intervention.
